# Determination of total flavonoids in three **Sedum** crude drugs by UV–Vis spectrophotometry

**DOI:** 10.4103/0973-1296.71784

**Published:** 2010

**Authors:** Yujie Chen, Jing Wang, Dingrong Wan

**Affiliations:** *Pharmacy College of South-Central University for Nationalities, Wuhan, Hubei Province – 430074, People’ Republic China*

**Keywords:** Genus **Sedum**, quality control, total flavonoid content, UV–Vis spectrophotometry

## Abstract

A simple, rapid UV–Vis spectrophotometry method for the determination of total flavonoids in **Sedum sarmentosum** Bunge., **S. lineare** Thunb., and *S. erythrostictum* Migo. was developed, with a good linearity, precision, and stability. The detection wavelength was set at 500 nm, and an extraction solvent was optimized. Through the comparative study of multiple samples of the three plant drugs, their collected seasons and the habitats can be preliminarily ascertained, which may help to control the quality of the medicines to some extent.

## INTRODUCTION

**Sedum sarmentosum** Bunge., **S. lineare** Thunb., and *S. erythrostictum* Migo. are three **Sedum** plant medicines widely used against hepatitis, dysentery, herpes zoster, and swelling in China. Since *S. sarmentosum* Bunge. is famous for its validity of acute or chronic hepatitis, it has been recorded by Chinese Pharmacopoeia (2010), and various preparations have been developed from it. According to the present reports, the active components of *S. sarmentosum* Bunge. due to liver protection and alanine aminotransferase (ALT) decrease mainly exist in the water portion and *n*-butanol portion which principally include the glycosides and flavonoids, suggesting that the medicinal properties of *S. sarmentosum* Bunge. might result from pharmacologically active bioflavonoids.[1–5] Thus, the total flavonoid determination could reflect the quality of the drug to some extent.

Traditional extraction techniques, such as maceration, heating reflux, and soxhlet extraction, are often effective but time consuming or labor intensive. In contrast, ultrasonic extraction can extract analytes from various matrices in a shorter time. Besides, the quantification of total flavonoids was usually completed by UV–Vis absorption at 500 nm and calculated against a stand reference in routine analyses.[6–9] Although a number of papers have reported on the quantification of total flavonoids in traditional herbal medicines, few described the determination combined with comparison among several kinds of herbal drugs from different habitats as well as various months of collection together.

This article aimed to develop a rapid simple approach to estimate the quality of the three crude medicines and try to preliminarily explore the relationships among the harvest period, habitat, and drug quality.

## MATERIALS AND METHODS

### Apparatus

KQ-500E ultrasonic apparatus (Kunshan Ultrasonic Instrument Co., Ltd., Beijing, People’s Republic of China) was applied here and the outpower was 500 W, with a frequency of 40 kHz. The determination was performed on a 757 CRT UV–Vis spectrophotometer (Lengguang Instrument Co., Ltd., Shanghai, People’s Republic of China).

### Reagents and materials

The standard rutin was purchased from National Institute for the Control of Pharmaceutical and Biological Products (batch number: 100080-200707); the ethanol and petroleum ether employed were of the analytical reagent grade. Double distilled water was used in this work.

The three plant medicines, harvested from Jianshi, Yichang, Huangmei, and Wuhan, Hubei province, in different months were air-dried immediately under 60°C and were respectively identified as *S. sarmentosum* Bunge., **S. lineare** Thunb., and *S. erythrostictum* Migo. by Professor Wan Dingrong from College of Pharmacy, South-Central University for Nationalities [Table 1].

**Table 1:** Drug samples and their sources

Species	Habitat	Date of	People for whom collection was done and specimen number
*S. sarmentosum* Bunge.	Jianshi	April 27, 2006	Congrong Wang 060427
		June 28, 2006	Congrong Wang 060620
		September 16, 2006	Congrong Wang 060901
	Yichang	April 16, 2006	Dingrong Wan 060401
		July 14, 2009	Yujie Chen, Jing Wang 090703
		September 29, 2009	Yujie Chen, Jing Wang 090906
	Wuhan	April 3, 2006	Dingrong Wan 060403
		June 6, 2009	Dingrong Wan 090601
		August 29, 2006	Dingrong Wan 060810
**S. lineare** Thunb.	Huangmei	April 2, 2009	Dingrong Wan 090401
	Luotian	July 14, 2006	Dingrong Wan 060706
	Huangmei	October 5, 2008	Jing Wang, Ran Xu 081001
*S. erythrostictum* Migo.	Jianshi	April 26, 2006	Congrong Wang 060427
		June 27, 2006	Congrong Wang 060627
		September 17, 2006	Congrong Wang 060908
	Wuhan	April 3, 2006	Dingrong Wan 060403
		May 2, 2006	Dingrong Wan 060505
		October 4,	Wan Dingrong
		2006	061004

### Sample preparation

Each dried plant drug was crushed and passed through a 20-mesh sieve. Then 1.0 g of each sample was accurately taken, mixed with 50 ml of petroleum ether, and extracted ultrasonically twice for 40 min. Poured out the petroleum ether solution. After the sample powder was dried completely, 100 ml of 70% ethanol was added, and subjected to another ultrasonic extraction for 90 min (3 × 30 min) at 55°C, and then filtered into a flask to make a total volume of 100 ml with 70% ethanol.

### Standard preparation

A total of 200.0 mg/l of the rutin standard solution was prepared by dissolving rutin reference material in 70% ethanol.

### Procedures for the determination of total flavonoids

A total of 4 ml of each sample extraction was pipetted into a 10-ml volumetric flask. The solution was treated with 0.40 ml of the 5% NaNO_2_ solution for 6 min and evenly mixed, into which 0.4 ml of the 10% Al(NO_3_)_3_solution was added and shaked up; then 6 min later, 4ml of the 4% NaOH solution was added to it. The mixture was diluted to the volume with double distilled water, and allowed to stand for 15 min before analyzing against the blank solution.

## RESULTS AND DISCUSSION

### Selection of the detection wavelength

The absorption spectra of the three sample extraction and rutin solutions were obtained. The absorption peak of rutin was at 510 nm and **S. lineare** Thunb. at 500nm, while both **S. lineare** Thunb. and *S. erythrostictum* Migo. solutions had strong absorptions at a little less than 500 nm. Comprehensively, 500 nm was chosen as the detection wavelength.

### Optimization of the extraction solvent

**Figure 1:**
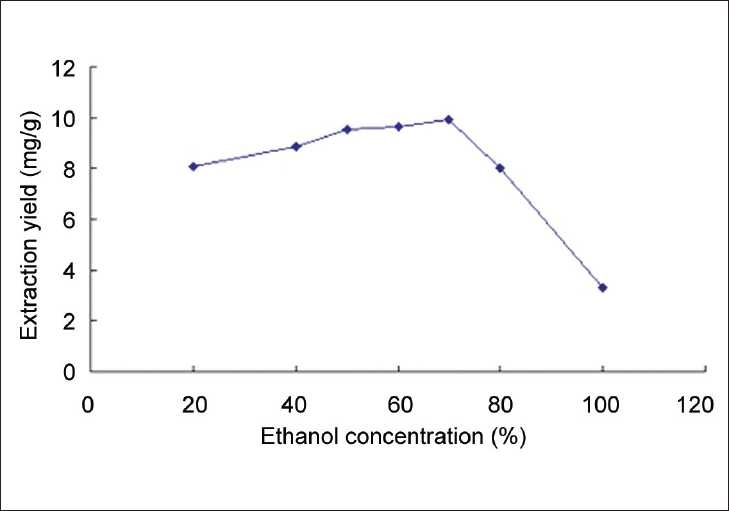
The relationship between the ethanol concentration and the flavonoid extraction yield of *S. sarmentosum* Bunge.

Since flavonoids contain some hydroxyl groups, they often dissolve easily in alcohol or alcohol–water mixtures. The *S. sarmentosum* Bunge. sample (Jianshi, June 28, 2006) was employed here, trying to find out the optimum solvent for sample extraction. The relationship between alcohol concentration and the yield of the total flavonoids is shown in Figure 1. In the figure, we could see that the extraction yield kept increasing and reached the top when the ethanol concentration went up to 70%, and then began to fall sharply as the ethanol concentration continued growing. Hence, the optimum extraction solvent turned out to be 70% ethanol.

### Method validation

All the sample solutions applied here were made from the *S. sarmentosum* Bunge. sample collected in Jianshi, June 28, 2006.

The linearity of the method was tested by analyzing different amounts of the standard solution (0, 0.2, 0.5, 1, 2, 3, 4 ml, respectively) coupled with isometric color development reagents according to the procedures for the determination of total flavonids. A good linear response was shown (*r* > 0.999) over the range of 4–80 μg/ml, with an equation *A* = 12.155C + 0.005, in which A meant the absorbance value while C (mg/mL) represented the concentration of the rutin solution.

Exactly took 4 ml of the *S. sarmentosum* Bunge. sample solution, operated according to the colorimetric method mentioned above and determined successively for five times. The average absorption was 0.489, and the RSD attained was 0.09%, demonstrating that the instrument used had a high precision.

A series of *S. sarmentosum* Bunge. samples were taken and treated according to sample preparation and colorimetric methods mentioned above. The flavonoid contents calculated from regression equation were 1.06%, 1.05%, 1.01%, 1.04%, 1.01%, and 1.00%, so the average content was 1.03% with RSD < 2.7% (*n* = 6), investigating a good repeatability.

Recovery experiment was performed to evaluate the accuracy of the methods. 1.0g of *S. sarmentosum* Bunge. sample powder were spiked with 0.5ml rutin solution (containing 10 mg of rutin) prior to the extraction. The spiked samples were analyzed in six copies. Recoveries of total flavonoids obtained are shown in Table 2, which informed us that the method possessed a nice accuracy.

**Figure 2:**
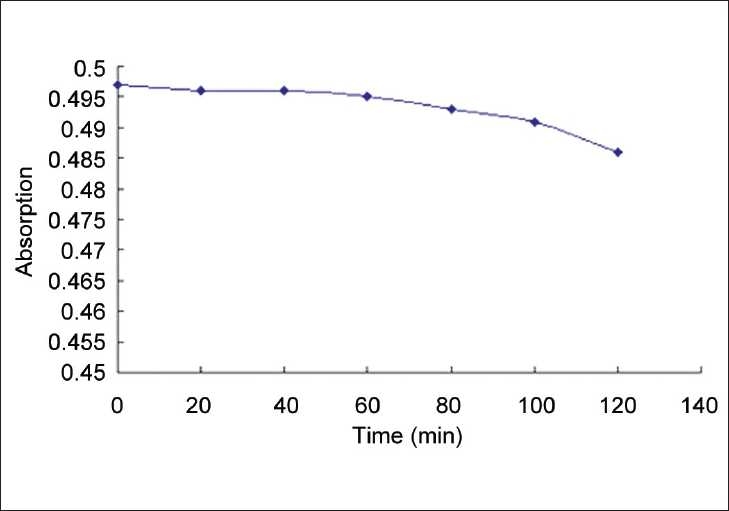
The relationship between the analysis time and absorption

After the extraction of *S. sarmentosum* Bunge., the sample mentioned above was dealt with according to the procedures for the determination of total flavonoids; absorptions were measured every 20 min ranging from 0 min to 120 min [Figure 2]. It’s clear that the absorption of the solution was relatively stable (RSD = 0.78%) if the measurement was carried out within 120 min. Thus, all the analyses should be performed within 2 h after the color reaction.

**Table 2:** Recoveries of total flavonoids

Sample amount (g)	Flavonoids in the sample (mg)	Spiked rutin amount (mg)	Determined flavonoid amount (mg)	Recovery (%)	Mean recovery (%)	RSD
1.0003	10.3031	10	20.07404	0.977094	93.8	2.80
1.0003	10.3031	10	19.7861	0.9483		
1.0002	10.3021	10	19.86837	0.956627		
1.0001	10.3010	10	19.58042	0.927942		
1.0001	10.3010	10	19.49815	0.919715		
0.9999	10.2990	10	19.29247	0.899347		

### Sample analysis

The present method was applied to the analyses of flavonoid contents in three crude drugs from Genus **Sedum** and the results are displayed in Table 3. It is obvious that all the related plant drugs contain flavonoids, which may be associated with their common pharmacological actions.

**Table 3:** Total flavonoids in three crude drugs from the genus **Sedum**

Species	Habitat	Date of collection	Flavonoid content (%)
*S. sarmentosum* Bunge.	Jianshi	April 27, 2006	2.09
		June 28, 2006	1.04
		September 16, 2006	1.41
	Yichang	April 16, 2006	2.52
		July 14, 2009	1.10
		September 29, 2009	1.22
	Wuhan	April 3, 2006	1.93
		June 6, 2009	0.82
		August 29, 2006	0.64
**S. lineare** Thunb.	Huangmei	April 2, 2009	1.88
	Luotian	July 14, 2006	0.91
	Huangmei	October 5, 2008	1.44
*S. erythrostictum* Migo.	Jianshi	April 26, 2006	2.18
		June 27, 2006	1.10
		September 17, 2006	0.85
	Wuhan	April 3, 2006	1.04
		May 2, 2006	0.86
		October 4, 2006	0.66

**Figure 3:**
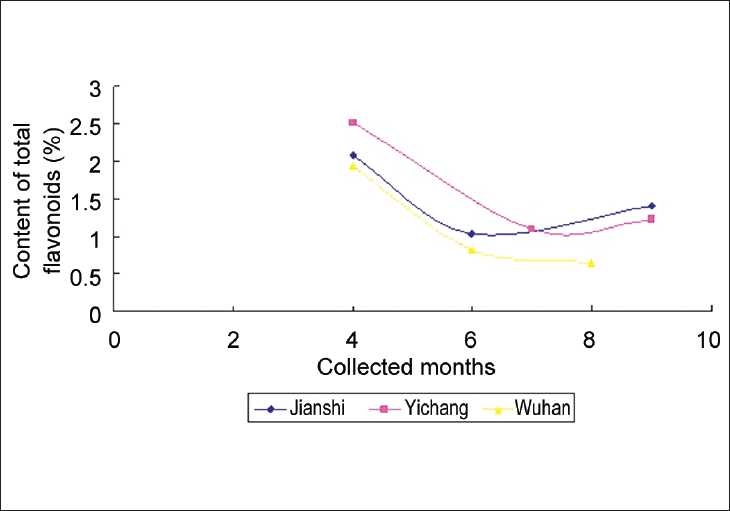
The relationship among month of collection, habitat and the content of total flavonoids in *S. sarmentosum* Bunge.

**Figure 4:**
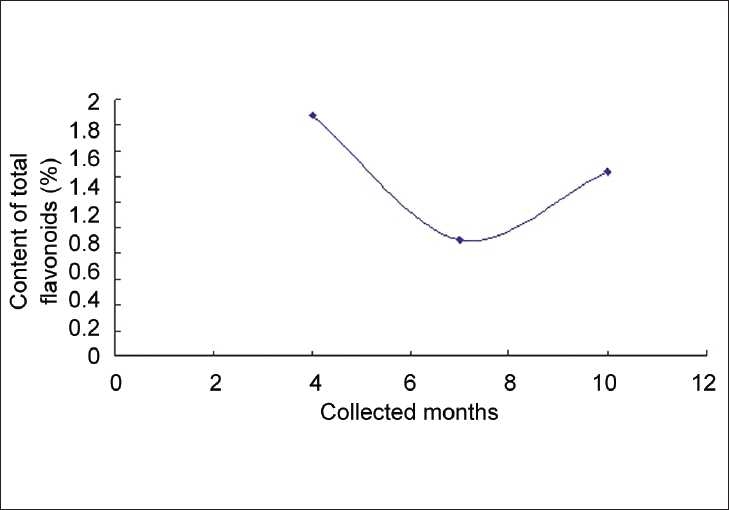
The relationship between month of collection and the content of total flavnoids in **S. lineare** Thunb

**Figure 5:**
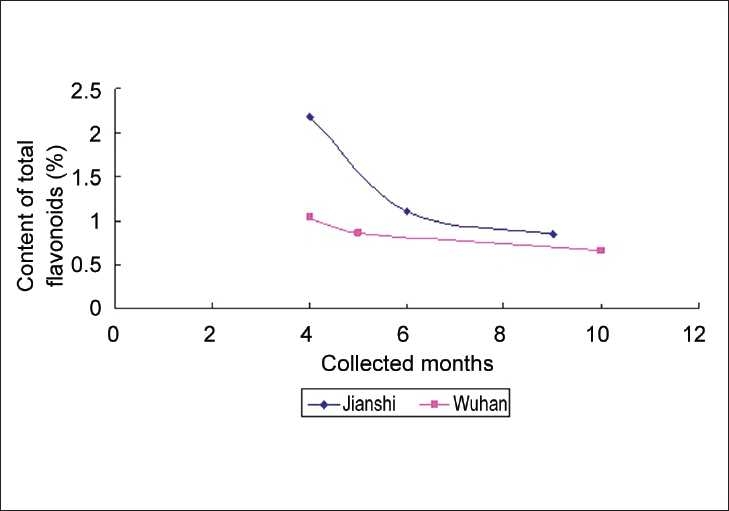
The relationship among month of collection, habitat and the content of total flavnoids in *S. erythrostictum* Migo

A relationship between total flavonoid content and harvest season is also revealed by Table 3. All the three plant medicines contained maximum flavonoids in April (flowering period), and then the flavonoid quantities kept decreasing from April to June or July (during this time, the leaves of *S. erythrostictum* Migo. mostly fell down). But for *S. sarmentosum* Bunge. and **S. lineare** Thunb., the contents began to rise a little after August [Figures 3 and 4] (except *S. sarmentosum* Bunge. sample collected in Wuhan), while the flavonoid quantities of *S. erythrostictum* Migo. continued to drop during autumn [Figure 5]. It seemed that all the three plant drugs should have the best quality if harvested in flowering time. Thus, the quality could be preliminarily controlled by flavonoid determination.

On the other hand, it was also manifested that a certain relation existed between the flavonoid content and the sample habitat. Both Figures 3 and 5 informed us that samples collected in the west of Hubei Province (Jianshi or Yichang) usually possessed higher flavonoid quantities than those collected in the east. Therefore, it was reasonable to deduce that Western Hubei should be a more suitable habitat to collect the three crude drugs from genus **Sedum** than the east.

## CONCLUSION

As the components contained in a Chinese traditional medicine are various and complicated, the strong polar glycosides, i.e., *S. sarmentosum* Bunge. glycoside (Sarmentosin), are not the only active composition associated with hepatitis treatment. Flavonoid ingredients are also the key point. Therefore, the flavonoid quantities reflected the quality of the three medicines from one aspect.

Studies on total flavonoid determination of the *S. sarmentosum* Bunge., **S. lineare** Thunb., and *S. erythrostictum* Migo. samples collected from different habitats and months clearly manifested that the flavonoid contents are closely related to the collected seasons, which may help to judge the optimum harvest time of the three plant drugs, so that the quality could be preliminarily controlled. The results also suggested that the flavonoid content changed with the habitat. The samples harvested in the west of Hubei usually possessed higher flavonoid contents compared with those collected in the east.

The method established for the flavonoid determination in three crude drugs of genus **Sedum** was simple, direct, and accurate, providing a valuable reference for quality control.
